# Analysis of vertical ground reaction force variables by Foot scan in hemiplegic patients

**DOI:** 10.1186/1757-1146-7-S1-A98

**Published:** 2014-04-08

**Authors:** HyunDong Kim, Geun-Yeol Jo, NaMi Han, Mi-Ja Eom

**Affiliations:** 1 Department of Physical Medicine and Rehabilitation, Inje University, Busan, 614-735, South Korea; 2 Department of Physical Medicine and Rehabilitation, Inje University, Busan, 612-896, South Korea

## Purpose

Our purpose was to analyze the differences in the vertical ground reaction force (GFR) records of hemiplegic patients to a normal healthy control group and between affected and unaffected limbs of hemiplegic patients using an F-Scan in-shoe transducer.

## Material and methods

Twenty patients with hemiplegia due to vascular causes underwent gait analysis (by the F-Scan system). All patients had steady neurological status and were able to gait independently more than 8 m. The following vertical ground reaction force variables were measured: the first peak force during early stance(F1Y) and the percent stance at which it occurred(F1X); the second peak force evident through push-off(F2Y) and the percent stance that it occurred(F2X); loading rate, push-off rate, vertical force impulse, and stance time. Comparison of the GFR records was performed between hemiplegic patients and healthy control group and affected and unaffected limbs of hemiplegics. The group control consisted of 20 healthy volunteer subjects.

## Results

F1Y of affected side was significantly less than that of unaffected side and normal control group(p<0.05). There was no difference in the F2Y between affected side and control group but that of unaffected side was significantly less than the other groups(p<0.05). F1X of both affected and unaffected side of patients was greater and F2X was less than the control group(p<0.05). Loading rate and push-off rate were significantly less on the both affected and unaffected sides of patients when compared to the control group(p<0.05). Greater impulse and stance time were recorded in both sides of patients than the control group(p<0.05).

## Conclusions

Measuring vertical ground reaction force variables using a F-scan system is an objective and useful way to analyze hemiplegic gait after stroke. From this study, we identified abnormal GFR variables in affected limb and even on unaffected limb of hemiplegic patients. Abnormalities in GFR variables of the unaffected limb in hemiplegic patients may not be the principle target of rehabilitation programs aiming at restoring gait pattern. Instead it is suggested that more account should be taken to the unaffected limb

